# Decoding the Chronic Obstructive Pulmonary Disease (COPD) Puzzle: Investigating the Significance of Exacerbation Scores in Triage Decision-Making

**DOI:** 10.7759/cureus.41975

**Published:** 2023-07-16

**Authors:** Nishant Allena, Sneha Khanal, Abhishrut Jog, Maria J Duran, Sujeirys Paulino, Srikaran Bojja, Maryam Soliman

**Affiliations:** 1 Pulmonary Medicine, BronxCare Health System, Bronx, USA; 2 Internal Medicine, BronxCare Health System, Bronx, USA; 3 Internal Medicine, Bronx Care Health System, Bronx, USA; 4 Pulmonary and Critical Care Medicine, BronxCare Health System, Bronx, USA

**Keywords:** risk scoring systems, prognostic scoring system, triage, acute exacerbation of chronic obstructive pulmonary disease, copd: chronic obstructive pulmonary disease

## Abstract

Chronic obstructive pulmonary disease (COPD) is a complex disease pathology of the lungs that has a significant impact on global health. It has been a major contributor to global mortality and morbidity, with COPD exacerbations posing a substantial economic burden on the healthcare systems. Appropriate triaging of patients with COPD exacerbation is crucial to reduce the burden of hospitalization, especially in the intensive care unit (ICU). Understanding the significance of exacerbation scores in triage decision-making is essential for improving outcomes and optimizing patient care. To aid this triage decision-making, several scoring systems have been developed. This review article aims to discuss the different scores, including assessment of Confusion, Urea, Respiratory rate, Blood pressure, and Age (≥65 years) (CURB-65); Dyspnoea, Eosinopenia, Consolidation, Acidaemia and atrial Fibrillation (DECAF), Neutrophil to lymphocyte ratio (NLR); Platelet-lymphocyte ratio (PLR); Pneumonia severity index/Pneumonia Patient Outcomes Research Team (PSI/PORT); and elevated BUN, Altered mental status, Pulse, Age (>65 years) (BAP-65), and their role in triaging COPD exacerbations. Proper triaging allows for the appropriate allocation of resources and timely interventions based on severity. Further research and validation are needed to establish the optimal use and integration of these scores in clinical practice, particularly in ICU settings.

## Introduction and background

Global Initiative for Chronic Obstructive Lung Disease (GOLD) 2023 defines Chronic Obstructive Pulmonary Disease (COPD) as a heterogeneous lung condition characterized by chronic respiratory symptoms (dyspnea, cough, expectoration, and/or exacerbations) due to abnormalities of the airways (bronchitis, bronchiolitis), and/or alveoli (emphysema) that cause persistent, often progressive, airflow obstruction ​[1]. It has an enormous disease burden on global health, affecting about 300 million people or 4% of the world's population and claiming 3.23 million lives by 2019 ​[2,3]. COPD exacerbation itself is an independent predictor of mortality ​[4]. It has been estimated that about 25% of COPD patients require ICU admission during their disease, leading to a significant economic burden on healthcare, amounting to an estimated cost of 50 billion on the American healthcare system ​[5,6]. Given the overwhelming freight of COPD on all-cause hospitalizations and ultimately ICU admissions, it is imperative that proper triaging be done for patients admitted with COPD exacerbation to decide on the ICU level of care to reduce ICU burden. 

Many scoring systems have been developed to aid in clinical decision-making for physicians. This article aims to summarize and assess the effectiveness of these scores in appropriate triage. Among the currently available triage scores: assessment of Confusion, Urea, Respiratory rate, Blood pressure, and Age (≥65 years) (CURB-65); Dyspnoea, Eosinopenia, Consolidation, Acidaemia and atrial Fibrillation (DECAF), Neutrophil to lymphocyte ratio (NLR), Platelet-lymphocyte ratio (PLR), Pneumonia severity index/Pneumonia Patient Outcomes Research Team (PSI/PORT), and elevated BUN, Altered mental status, Pulse, Age (>65 years) (BAP-65) are some of the most commonly used, each of them with their own individual components contributing to the score.

## Review

Scoring systems

CURB-65

CURB-65 score was first introduced in 2002 as CURB criteria by Dr. Lim et al. as the clinical prediction criteria for mortality in patients with community-acquired pneumonia (CAP) ​[7]. The components of the score since its inception were later modified to include the age ≥65 years for scoring and are listed in the table below (Table 1).

**Table 1:** Parameters of CURB-65 scoring CURB-65 scoring [8] CURB-65: Confusion, Urea, Respiratory rate, Blood pressure, and Age (≥65 years); SBP: Systolic blood pressure; DBP: Diastolic blood pressure; mmol/l: millimoles per litre; mmHg: millimeters of Mercury

Parameters	0 point	1 point
Confusion (defined as a Mental Test Score of 8 or less, or new disorientation in person, place, or time)	Absent	Present
Blood Urea >7 mmol/l,	Absent	Present
Respiratory rate ≥30 per minute,	Absent	Present
Blood pressure (SBP <90 mm Hg or DBP ≤60 mm Hg)	Absent	Present
Age > 65 years	Absent	Present

Each component is allocated one point, resulting in a six-point score ranging from 0 to 5 associated with comparable mortality risks (Table 2). The score was validated as a predictor of 30-day mortality in patients with CAP, thus guiding the treatment options for each group. It was further concluded that the comprehensive sensitivity, as well as specificity of CURB-65, was comparable to other validated tools for assessing the severity of CAP including the mBTS (modified British Thoracic Society) and the Pneumonia Severity Index (PSI) ​[9].

**Table 2:** CURB-65 score, mortality risk, and recommended treatment options CURB-65: Confusion, Urea, Respiratory rate, Blood pressure, and Age (≥65 years); ICU: Intensive Care Unit

Score	Mortality risks	Treatment options
0-1	Low (1.5%)	Home treatment likely
2	Intermediate (9.2%)	Consider hospitalization for treatment
3 or more	High (22%)	Treatment in hospital as severe pneumonia with consideration for ICU admissions for scores 4-5.

The CURB-65 score has also been used to predict ICU mortality in the setting of CAP. Although no solid adaptation has been established on the acute exacerbation of chronic obstructive pulmonary disease (AECOPD) front, some studies have utilized CURB-65 in predicting mortality in patients admitted with AECOPD alone, without CAP [10,11]. Various studies have conflicting evidence regarding mortality prediction in CAP-COPD patients. There is evidence suggesting higher CURB-65 scores in patients with CAP-COPD ​[12]​ whereas other studies demonstrate the lack of significant association between COPD and increased mortality in hospitalized patients with CAP ​[13]. This, in itself, calls for further research to predict the increase in in-hospital deaths conclusively and the use of CURB-65 scores to predict the very increase in hospital and ICU settings.

DECAF

The DECAF score serves as a prognostic score to evaluate mortality in patients with acute exacerbations of COPD. Each component of the DECAF score is assigned a numerical value and the total score is calculated as follows: Dyspnea with one point if the dyspnea is classified as “5a” using the extended Medical Research Council Dyspnea (eMRCD) and 2 points if is classified as “5b” using the eMRCD score ​[14]. Other components include eosinopenia (0.05 x 10^9^/L), the presence of consolidation, moderate or severe acidemia, and atrial fibrillation and for each of these components, 1 point is added (Table 3). The maximum DECAF score is 6 ​[15].

**Table 3:** DECAF parameters and scores DECAF parameters and scores table [15] DECAF: Dyspnoea, Eosinopenia, Consolidation, Acidaemia and atrial Fibrillation; eMRCD: extended Medical Research Council Dyspnea classification

Variables	Score
Dyspnea	
eMRCD 5a (too breathless to leave the house unassisted but independent in washing and/or dressing)	1
eMRCD 5b (too breathless to leave the house unassisted and requires help with washing and dressing)	2
Eosinopenia (eosinophils <0.05×109/L)	1
Consolidation	1
Moderate or severe acidemia (pH <7.3)	1
Atrial fibrillation (including history of paroxysmal atrial fibrillation)	1

The DECAF score serves as a tool to risk-stratify patients with AECOPD. DECAF Scores of 0-1 indicate low risk, a score of 2 represents intermediate risk, and scores of 3-6 indicate a high risk of inpatient mortality in patients with COPD exacerbation. It has been used in different studies to classify patients as low-risk and suitable for de-escalation of treatment, early discharges, and home treatment, resulting in better utilization of hospital resources as well as identification of high-risk patients that benefit from a prompt escalation of treatment ​[14,16]. 

NLR

NLR has been used in the past as a prognostic factor in certain malignancies. In the setting of AECOPD, it is being studied as a prognostic and inflammatory marker ​[17]. The NLR is the ratio of serum absolute neutrophil count to serum lymphocyte count. There is no cut-off to define an elevated NLR ratio; however, one study defined it as “high NLR” a ratio ≥3.00 ​[16]. High NLR has been associated with the increased development of AECOPD. Research has suggested that stable elevated levels of NLR are more common in patients with AECOPD and that stable elevated NLR is an independent predictor for the development of AECOPD and COPD-related hospitalizations ​[18,19]​. While promising results are being published regarding NLR and its use as a prognostic marker in AECOPD, further studies are necessary to validate this score.

PLR

PLR, as its name implies, is the ratio of absolute serum platelet account to absolute serum lymphocyte count. This novel inflammatory marker is accessible and easy to conduct, as it can be measured with a simple Complete Blood Count (CBC). This has been proven helpful in predicting inflammation and mortality in conditions like cardiovascular events ​[20]​ and cancers ​[21]. 

A cut-off for PLR has not been established yet, but a study established that a PLR >235 was associated with increased 90-day mortality in patients admitted with COPD exacerbation ​[22]. In a retrospective study of 303 patients, the mean level of PLR in all patients with AECOPD was found to be 207.21±148.47, with higher levels in non-survivor patients ​[23]. The use of both NLR and PLR showed a significant association with 28-day mortality in AECOPD requiring hospitalization ​[24]. The usefulness of PLR as a predictive tool for AECOPD requires further investigation and validation, especially in ICU settings. 

PSI/PORT

The PSI/PORT scoring was developed as a prognostic tool and has been validated by a number of studies to predict the severity of pneumonia ​[25,26]. It has since been adopted for the severity prediction of AECOPD. This score comprises 20 variables, considering the patient's characteristics, coexisting illnesses, physical examination findings, and laboratory and imaging studies results. Each of these variables is assigned a numerical value in a stepwise approach as follows (Tables 4-5).

**Table 4:** PSI/PORT parameters and scoring system: Risk class stratification Class I vs Classes II-IV Pneumonia severity index/Pneumonia Patient Outcomes Research Team (PSI/PORT) parameters and scoring system table [8]

Step1: Risk class stratification- Risk class I vs Risk classes II-V		
Presence of:		
	Over 50 years of age	Yes/No
	Altered mental status	Yes/No
	Pulse ≥125 per minute	Yes/No
	Respiratory rate >30 per minute	Yes/No
	Systolic blood pressure <90 mm Hg	Yes/No
	Temperature <35 °C or ≥40 degrees Celsius	Yes/No
History of:		
	Neoplastic disease	Yes/No
	Congestive heart failure	Yes/No
	Cerebrovascular disease	Yes/No
	Renal disease	Yes/No
	Liver disease	Yes/No

If “No” to all the above parameters in Table 4 identify as Risk Class I; if “Yes” to any of the above parameters, then move to Step 2 (Table 5).

**Table 5:** PSI/PORT parameters and scoring system: Risk class stratification classes II-V Pneumonia severity index/Pneumonia Patient Outcomes Research Team (PSI/PORT) parameters and scoring system table [8] mg/dl: milligrams per deciliter; mmol/l: millimoles per liter

Step 2: Risk class stratification- Classes II-V		
Demographics		Points Assigned
	Male	+Age (years)
	Female	+Age (years) − 10
	Nursing home resident	+10
Comorbidity		
	Neoplastic disease	+30
	Liver disease	+20
	Congestive heart failure	+10
	Cerebrovascular disease	+10
	Renal disease	+10
Physical Examination		
	Altered mental status	+20
	Pulse ≥125 per minute	+10
	Respiratory rate >30 per minute	+20
	Systolic blood pressure <90 mm Hg	+20
	Temperature <35 °C or ≥40 degrees Celsius	+15
Laboratory and Radiographic Findings		
	Arterial pH <7.35	+30
	Blood urea nitrogen ≥30 mg/dl (9 mmol/l)	+20
	Sodium <130 mmol/l	+20
	Glucose ≥250 mg/dl (14 mmol/l)	+10
	Hematocrit <30%	+10
	Partial pressure of arterial O2 <60mmHg	+10
	Pleural effusion	+10

The total number of points will help sort our patients into five classes. Class I and Class II (<70 points) are low-risk patients that can be managed as an outpatient. For Class III (between 71 and 90 points), the physician can choose outpatient management versus a short observation period based on clinical judgment; Class IV (between 91 and 130 points) is a patient with moderate risk and needs inpatient admission. Lastly, Class V (>130 points) is considered high-risk and needs inpatient admission ​[27]. 

*BAP-65* 

BAP -65 is a risk stratification score used in AECOPD consisting of four components. Each component has been assigned one point with a score ranging from 0-4, classifying patients into five classes (Class I to Class V). Each of the parameters: BUN>25 mg/dl =Urea >53.5 mg/dl, altered mental status, pulse rate >109 beats per minute, and age >65 years account for one point each (Table 6) [28].

**Table 6:** BAP-65 parameters and score Elevated BUN, Altered mental status, Pulse, Age (>65 years) (BAP-65) parameters and score table [28] mg/dl: milligrams per deciliter

Parameters	0 point	1 point
BUN (blood urea nitrogen) > 25 mg/dl = UREA > 53.5 mg/dl	Absent	Present
Altered mental status (AMS)	Absent	Present
Pulse rate (PR) > 109 per minute	Absent	Present
Age > 65 years	Absent	Present

For patients who have none of the three main risk factors (BUN level ≥ 25 mg/dL, altered mental status, or pulse ≥ 109 beats/min), those ≤ 65 years of age are designated as class I, whereas patients with no risk factors who are > 65 years of age are classified as class II. The designation into risk classes III, IV, and V are based on whether the patient has one, two, or three of the central risk factors, respectively (Table 7). Early invasive ventilation or ICU admission was validated for Class III and above ​[29].

**Table 7:** BAP-65 risk class stratification BAP-65: Elevated BUN, Altered mental status, Pulse, Age (>65 years)

Class	BAP	Age
I	0	<65 years
II	0	≥65 years
III	1	Any age
IV	2	Any age
V	3	Any age

Discussion

The COPD exacerbation scoring systems take multiple variables into account as mentioned in our individual score descriptions above. Some of the common factors that have been observed among a majority of the scores are altered mental status, respiratory rate, age, BUN or renal function, and pulse rate. The PSI/PORT and DECAF scores are the only scoring systems to include acidemia (< 7.35 and < 7.30 respectively) and radiological findings as variables. The efficacy of each score has been studied in various trials. 

Beginning with CURB-65, a retrospective study done in patients with COPD revealed a significantly high in-hospital mortality of 80% in patients with CURB-65 scores of 3 or more compared to scores of 0-1 or 2 that showed 10% mortality in each group respectively ​[30]. DECAF score takes into account atrial fibrillation as a variable. DECAF score has been concluded by validation studies to be a strong predictor of mortality and given the reliability and accuracy to risk-stratify patients ​[31]. Atop that, the presence of atrial fibrillation has also been established to be an independent risk factor of a significant increase in both all-cause mortality as well as cardiac mortality in patients with COPD ​[32,33].

On the other hand, the NLR score has been suggested to be a predictor of inflammation in COPD, associated with higher levels during acute exacerbations and increased severity of exacerbations ​[34,35]. NLR levels ≥ 2.8 were associated with increased odds of hospitalization in AECOPD patients ​[36]. In a retrospective study of 1704 patients hospitalized with COPD, an elevated NLR cut-off of ≥ 7 was found to be associated with increased 6-month mortality, indicating its potential use as a guide for treatment ​[37]. However, more research is needed to validate NLR as a biomarker for predicting mortality in AECOPD. PLR has been studied as a predictive indicator for patients with acute exacerbation of COPD. A study found an increase in PLR in patients with severe airway limitation and GOLD stages C and D compared to mild to moderate airway disease. Studies have demonstrated that both NLR and PLR could be used as prognostic biomarkers of mortality in hospitalized patients with AECOPD, with NLR demonstrating better sensitivity and specificity as compared to PLR [23,24]. In addition, the combination of NLR and PLR has been shown to be more accurate than either alone [38].​

In a cohort study titled "The Pneumonia Severity Index as a Predictor of In-Hospital Mortality in Acute Exacerbation of Chronic Obstructive Pulmonary Disease," Hu et al. aimed to determine if the PSI could predict in-hospital mortality for patients with AECOPD and compared its efficacy with that of the CURB65 and BAP65 indexes [39]. The study included 752 patients admitted with COPD exacerbation at the Affiliated Hospital of Guangzhou Medical University, Guangzhou, China, from July 2010 to May 2014, and each patient was assessed using the PSI, CURB-65, and BAP-65 scores. The findings revealed that the PSI score had excellent discriminative ability for in-hospital mortality, with an area under the curve (AUC) of 0.860 (95% confidence interval {CI} = 0.816-0.903). Additionally, the adoption of PSI to AECOPD was proven as a superior predictor than CURB-65 or BAP-65 for in-hospital mortality in hospitalized patients ​[39]. BAP-65 can be a quick and easy tool for AECOPD severity prediction. Studies have demonstrated that BAP-65 class III or higher has been associated with an increased need for mechanical ventilation and higher mortality in COPD exacerbations ​[40]. However, a validation study done to assess the BAP-65 score to stratify patients on the risk of poor in-patient outcomes showed that the score had inadequate accuracy for such prediction ​[41]. Comparative studies have been conducted between various scoring; however, further research is mandated on the BAP-65 front given its utility in risk prediction, particularly in resource-limited settings. 

A comparative systematic review between DECAF and CURB-65 scores established that the DECAF score had more promising accuracy in predicting in-hospital and 90-day mortality, while CURB-65 better predicted 30-day mortality in AECOPD patients ​[42]. A prospective study by Gayaf et al. in 2021, titled "Which one is superior in predicting 30 and 90 days mortality after COPD exacerbation: DECAF, CURB-65, PSI, BAP-65, PLR, NLR," included 141 patients admitted for AECOPD and compared various scores to predict mortality, and established for each scoring system, higher scores were associated with increased mortality [43]. However, of all the scoring systems evaluated, CURB-65 was slightly superior in predicting mortality at 30 and 90 days after AECOPD, with odds ratios of 2.968 and 2.284, respectively, and 95% CI 1.264-6.971 at 30 days and 1.125-4.637 at 90 days ​[43]. 

## Conclusions

COPD exacerbation mortality prediction scores serve as a useful tool to estimate the need for possible ICU admission. Overall, while no single score can fully capture the complexity of COPD, using these tools in combination with clinical judgment may help improve patient outcomes and optimize treatment plans. Our review concludes that more studies, including meta-analyses, comparing the COPD scoring systems need to be done to establish guideline-directed prediction of AECOPD severity and mortality with a single scoring system or a combination of many.
